# The Knowledge and Awareness for Radiocesium Food Monitoring after the Fukushima Daiichi Nuclear Accident in Nihonmatsu City, Fukushima Prefecture

**DOI:** 10.3390/ijerph15102289

**Published:** 2018-10-18

**Authors:** Nobuaki Kunii, Maya Sophia Fujimura, Yukako Komasa, Akiko Kitamura, Hitoshi Sato, Toshihiro Takatsuji, Masamine Jimba, Shinzo Kimura

**Affiliations:** 1 Laboratory of International Epidemiology, Fukushima Branch Office, Center for International Cooperation, Dokkyo Medical University, Tochigi 321-0293, Japan; hjjqs457@ybb.ne.jp (N.K.); yukakokomasa@gmail.com (Y.K.); 2 Department of Community and Global Health, Graduate School of Medicine, The University of Tokyo, Tokyo 113-0033, Japan; mayafujimura@yahoo.com (M.S.F.); akikokitamura@gmail.com (A.K.); mjimba@m.u-tokyo.ac.jp (M.J.); 3 Department of Radiological Sciences, Ibaraki Prefectural University of Health Sciences, Ibaraki 300-0394, Japan; satoh@ipu.ac.jp; 4 Graduate School of Fisheries and Environmental Sciences, Nagasaki University, Nagasaki 852-8521, Japan; takatsuj@nagasaki-u.ac.jp

**Keywords:** radiation, food monitoring, Fukushima

## Abstract

On 11 March 2011, Japan experienced a massive earthquake and tsunami that triggered the Fukushima Daiichi Nuclear Power Plant (FDNPP) accident, resulting in the release of large amounts of cesium-134 and -137 into the atmosphere. In addition to the food radioactivity control in the markets throughout the country, radiocesium concentrations in locally grown foods were voluntarily inspected and the results were shown to the residents by the local government to raise their awareness of the internal radiation contamination risk from low knowledge in Nihonmatsu City, Fukushima Prefecture. In this longitudinal study, local food products for in-home consumption were evaluated by seven different food radioactivity measuring devices in Nihonmatsu City from 2011–2017. Radiocesium was detected in local foods in Nihonmatsu City even six years after the FDNPP accident. The highest number of products tested was in 2012, with the number steadily decreasing thereafter. Most foods had contamination levels that were within the provisional regulation limits. As edible wild plants and mushrooms continue to possess high radiocesium concentrations, new trends in radioactivity in foods like seeds were discovered. This study highlights that the increased risk of radiation exposure could possibly be due to declining radiation awareness among citizens and food distributors. We recommend the continuation of food monitoring procedures at various points in the food processing line under the responsibility of the government to raise awareness for the reduction of future risks of internal exposure.

## 1. Introduction

### 1.1. Fukushima Daiichi Nuclear Power Plant and the Measures Taken

On 11 March 2011, Japan experienced a massive earthquake and tsunami that triggered the Fukushima Daiichi Nuclear Power Plant (FDNPP) accident. This resulted in the release of large amounts of radionuclides into the atmosphere, including cesium-134 and -137 (^134^Cs and ^137^Cs, respectively) [1]. The radiation then spread across northeastern and eastern Japan, making radioactive contamination a significant societal problem, with radiocesium still detected in agricultural products in the affected areas. Therefore, it is critical to continue monitoring and measuring radioactivity levels to ensure the safety and health of Fukushima Prefecture residents.

On 17 March 2011, the Ministry of Health, Labor and Welfare of Japan released a provisional regulation limit of 500 Bq/kg for radiocesium in grains, vegetables, meat, and fish. However, on 1 April 2012, the limit was lowered to 100 Bq/kg for all foods, excluding infant food, milk, water, and beverages [2]. Local governments in Fukushima were required to perform surveys weekly or more frequently when radioactivity levels were close to the provisional limits [3]. Widespread contamination of the human food chain was avoided, and there has been no evidence of serious risks to health from radiation [4]. However, this does not eliminate the possibility of future risks of internal radiation exposure from food products.

### 1.2. Health Effects of Radiation

General knowledge on radioactivity in foods has been garnered from previous nuclear accidents, such as the 1986 Chernobyl disaster. High internal levels of ^137^Cs, which are indicative of radiation poisoning, can occur through ingestion of contaminated foods and can increase cancer risk [5]. Physical effects from radiation exposure were issues in past radiation accidents such as Chernobyl, including psychological and social effects like clinical depression and anxiety disorders [6]. Following the Chernobyl disaster, humans and domestic animals close to the nuclear plant were diagnosed with high internal radiation exposure resulting from ingestion of crops grown in radiocesium-contaminated soil [7]. Even 30 years after the disaster, wild mushrooms from Chernobyl still contained a high concentration of radiocesium, reflecting long-term environmental damage [8].

Internal radiation exposure and food safety are major causes of anxiety in Fukushima residents; indeed, the risk of exposure is considerable given the long half-life of ^137^Cs (30.2 years as compared to 2.06 years for ^134^Cs) [9]. Seasonal fluctuations in agricultural practices and food availability can also lead to varying radionuclides levels, causing a concern in food safety [10,11]. In the case of the western part of Bryansk Oblast, Russia, area affected by the Chernobyl disaster, consumption of mushrooms and wild berries containing high levels of Cs was tended to increase in the summer and autumn [12], and radiocesium concentrations were higher during these seasons in Japan as well [13].

This is of concern because residents of this region typically grow their own food for several reasons, such as in-home consumption, gifting, and foraging. Although the detection rate of radiocesium is decreasing annually due to the natural decay of radionuclides, certain foods consistently exhibit radioactivity levels over the provisional regulation limit. The accumulation of radiocesium in locally grown foods can potentially lead to adverse health effects in consumers.

### 1.3. Knowledge and Awareness of Radiation among Citizens

Multiple cases from inside Fukushima, as well as neighboring prefectures, such as Ibaraki, Niigata, Yamagata, and Gunma, have reported exceeding the standard value of radioceisum among wild edible plants sold at roadside farmer’s markets, even five or more years after the accident [14,15,16]. In May 2018, Fukushima City announced that 130 wild edible plants exceeding the standard value (100 Bq/kg) radioactive cesium were detected from a fruit and vegetable distributor in the city. A total of 254 packs had already been shipped to five locations in the prefecture, such as local farmer’s markets [17]. Another incident in May 2016, radioactive cesium of 234 Bq/kg, exceeding the reference value, was detected from bamboo shoots served in school lunches at an elementary school in Utsunomiya City, Tochigi Prefecture, located 141km southwest from the nuclear plant. According to the food distributor, sample items were collected without knowing it was from a restricted area [18].

### 1.4. Local Radiation Efforts

Nihonmatsu City is one of the 59 cities in Fukushima Prefecture, which is located 35–70 km from the FDNPP. Unlike other cities in the prefecture, the region contains high levels radiation, but residents were not ordered to evacuate by the government. Therefore, Nihonmatsu City has been carrying out various activities to reduce the risk of radiation exposure, such as ongoing decontamination of neighborhoods, schools, parks, and agricultural fields. In addition, elementary and junior high schools participate in an annual lecture taught by a radiation expert on the implications of living with low levels of radiation, potential health effects of contaminated foods, and ways to avoid external and internal radiation exposure. Radiation seminars by the experts are held once every two months for residents who express interest in learning about radiation, as well as voicing anxiety, concerns, and questions. The city annually measures external radiation exposure of residents from newborns to junior high school students, as well as some high school students and adults, across a 2-month period [19]. Furthermore, internal exposure inspections have been implemented among the residents in various ways. 

Since it is customary for residents of Nihonmatsu City to eat home-grown plants, such as vegetables, edible wild plants, and mushrooms, radiocesium concentrations of products can be monitored at several locations across the city to identify potentially hazardous foods for residents. Residents living in mountain areas are more likely to eat home-grown foods than the urban residents. In particular, a common pastime for older adults in the area is to forage for edible wild plants in the mountains or hunt for wild animals. 

The present study investigated radiocesium concentrations in home-grown foods to determine the risk of internal radiation exposure to residents of Nihonmatsu City, Fukushima Prefecture, and to raise their awareness of the risk associated with low knowledge in radiation contamination.

## 2. Materials and Methods 

In this longitudinal study from November 2011 to December 2017, radiocesium levels in agricultural products, such as vegetables, grains, and fruits were measured using food radioactivity measuring devices installed at Nihonmatsu City Hall, city branch offices, and resident centers. These food samples were voluntarily brought in by the residents of Nihonmatsu City, for their home consumption. The products inspected at the locations strictly prohibited measuring products which were intended for sale or for restaurant use [20].

Foods were categorized into 10 different groups, including potatoes, vegetables, grains, beans, fruits, seeds, edible wild plants, mushrooms, processed products, and “other”, which consists of miscellaneous foods such as meats, eggs, dairy, seaweed, and beverages. Owing to the emergency situation, samples were collected on a case-by-case basis at the beginning of the survey period, rather than in a systematic manner.

Food products were inspected at 18 different locations across the city by trained municipal staff. All monitoring locations were available for inspection five days a week from 8:30 a.m. to 5:15 p.m. As of April 2018, Nihonmatsu City scaled back the number of locations to seven locations where residents could receive inspection for their food samples.

Radioactive ^134^Cs and ^137^Cs concentrations were measured with a single instrument at each location, except for nine instruments at the main location, the Head Office of Radioactive Substance Measurement Center [21]. The following seven instruments were used: CAN—OSP—NAI (Hitachi—Aloka Medical, Mitaka, Japan); CAPTUS-3000A (ACROBiosystems, Zhuhai, China); FF1 (Nichei Industry, Fukushima, Japan); FD-08Cs1000-1C (Techno-X, Osaka, Japan); AFT-NDA2 (Advanced Fusion Technologies, Lihue, HI, USA); LB2045 (Berthold Technologies, Bad Wildbad, Germany); and AT1320 (ATOMTEX, Minsk, Belarus). The method of food inspection varied according to the measuring instrument. There were two types of instruments: the first measured samples after finely grinding them into a powder that filled a 1-L plastic container, and the second type measured samples in their natural state. Both types of instruments required at least 700 g of the food sample.

The contamination measurements were reported immediately to the resident after the inspection. The city also reported the measurements of the home-grown agricultural products from each of the locations every month to their website, including the concentrations of ones exceeding the provisional regulation limit. 

Data were analyzed using Microsoft Excel 2016 software (Microsoft, Redmond, WA, USA). The study was conducted with the cooperation of the local government of Nihonmatsu City. This work was supported by JSPS KAKENHI Grant Numbers JP25257504, JP24248060, JP17K19820. 

## 3. Results

### 3.1. Summary of Food Samples Collected

Local foods produced for personal consumption were inspected for radiocesium contamination. Every sample was voluntarily brought into one of the centers for inspection. The foods were categorized into 10 food groups which included various types of potato, vegetables, grains, beans, fruits, seeds, edible wild plants, mushrooms, processed products, and other miscellaneous foods, such as meats, eggs, dairy, seaweed, and beverages. Provisional regulatory radiocesium levels are 20–100 Bq/kg. These levels are divided into four categories: <20 Bq/kg (considered as not detected), 20–50, 50–100, and ≥100 Bq/kg. 

Table 1 shows the summary of the various categories of foods that were analyzed between 2011 and 2017. A total of 1672 food samples were tested in 2011 (over a 2-month period); 19,063 in 2012; 11,238 in 2013; 7521 in 2014; 7288 in 2015; 5997 in 2016; and 4194 in 2017. The number of tested foods steadily decreased each year after the maximum number in 2012. Out of the food categories, vegetables consist of the largest proportion of food samples collected annually, ranging from 31–51%.

### 3.2. Radiocesium Detection Rates by Food Group

Table 2 and Supplementary Figure S1 shows the radiocesium levels of each type of food from 2011 to 2017. Over a 6-year period, radiocesium levels exceeding 100 Bq/kg were recorded most frequently among mushrooms; over 50% of mushrooms had levels over 100 Bq/kg each year, except in 2017. In contrast, the levels in potatoes exceeded 100 Bq/kg only once during 2013. Radioactivity in potatoes was undetectable or <20 Bq/kg in 90% or more of samples each year, specifically 99% of the samples across the last four years. Other food categories such as vegetables, grains, beans, and fruits had undetectable levels of radiocesium, at least 90% of the time since 2015. However, a different trend was observed in seeds such as chestnut and ginkgo; where despite an initial decrease in seed samples with radiocesium levels >100 Bq/kg, an increase in radioactivity has been recorded since 2014. Edible wild plants are considered high-risk radioactive foods, since they typically grow in contaminated mountainous areas. As such, high radiocesium concentrations were detected in edible wild plants each year. The “Others” category contained a variety of samples such as meat, honey, and eggs. Nearly one-third of the inspected samples consisted of concentrations exceeding 100 Bq/kg. The “Total” indicates the total percentage in each detection rate of all products across six years. Potatoes showed the highest percentage of <ND products (98%), whilst mushrooms had the highest percentage of 100 Bq/kg < products (75%) in total.

### 3.3. Radiocesium Detection Rates by Food Group

**Figure 1:**
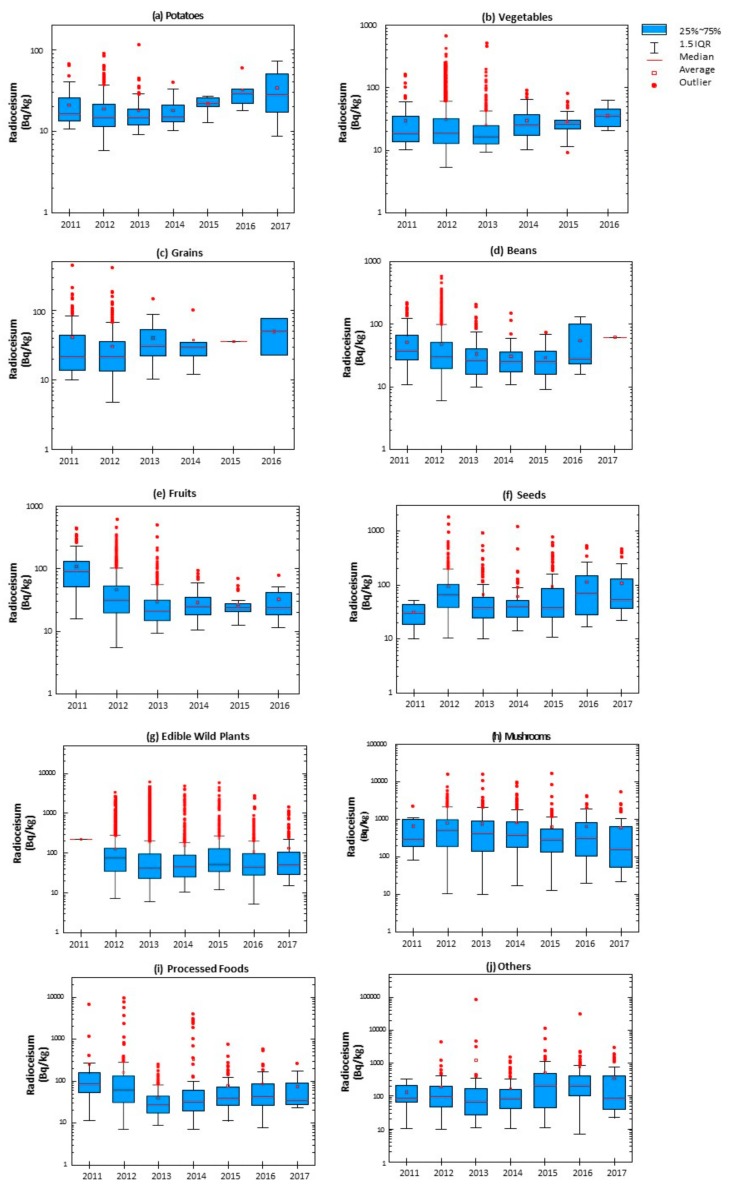
Boxplot of the radiocesium concentration of each year by food group: (**a**) Potatoes, (**b**) Vegetables, (**c**) Grains, (**d**) Beans, (**e**) Fruits, (**f**) Seeds, (**g**) Edible Wild Plants, (**h**) Mushrooms, (**i**) Processed Foods, (**j**) Others.

Figure 1 shows the distribution and variability in radiocesium concentration of each type of food from 2011 to 2017. The annual trend of the concentration across each food group significantly varied by food group, as well as each year. The boxplots use a logarithmic scale due to the skewness towards large values. 

Potatoes, vegetables, grains, beans, fruits (except in 2011) have consistently averaged <50 Bq/kg, below the regulation limit, each year. In addition, the number of outliers and the maximum values of these categories have shown a steady decrease since 2012.

On the contrary, out of the food categories, mushrooms resulted in the highest average radiocesium concentration each year. Other categories consistently detected over the regulation limit were edible wild plants and “others”. Additional trends have been observed among seeds. Seeds have shown an increase in the annual average radiocesium concentration since 2014. 

## 4. Discussion

From this study, the findings illuminate the level of awareness and caution towards radiation, and monitoring food contamination has been declining among the residents over the years since the accident.

The declining trend of inspected foods at the local monitoring centers could be a result of the fading awareness and perceived relevance of radiation risk, which decreased over time across the residents. The media, such as newspapers and TV, play a significant role in providing information to the citizens about food safety and risk perception. However, as time passes, less media reported on the situation in Fukushima and food safety. It is brought to the spotlight only when the radiocesium concentration surpasses the prohibitory level. The declining concern may also be due to increased recognition of the radioactivity in certain foods, such as mushrooms, edible wild plants, and certain meats. Through media or other sources, residents gained ability to recognize potentially contaminated agricultural products. In addition, a fraction of the residents who were more cautious of food safety, such as seniors and mothers with young children, may become the majority of the population who are more inclined to monitor their food products and utilize the food monitoring service. Nevertheless, close monitoring of radiation levels in home-grown foods, especially those consumed by children and pregnant women, is important for all residents [22]. 

Not only the residents’ level of awareness of radioactivity, but this trend may also be consistent among the awareness of food shippers and distributors. In relation to prefectures outside of Fukushima, some contaminated foods have been found to be exceeding the regulatory limit, which were found in the areas where monitoring systems were not set up [23]. In Yamagata Prefecture, located next to Fukushima, its Yamagata Newspaper reported in 2017 and 2018 that wild edible plants exceeding the standard value were collected from 5 towns and sold at roadside farmer’s markets located 112–174 km from the nuclear plant, as well as outside the prefecture [24,25,26].

By monitoring the concentration of food products across the years, the annual median, average, and outlier radiocesium values indicate which categories are a high-risk for food safety. One of the examples of which are seeds. Seeds have not been considered a high-risk radiation food group in past studies, but from the findings of this study, it can be considered as a high-caution food group. In the present study, seeds such as chestnuts and gingko initially showed a steady decrease in radiocesium concentration, in agreement with previous studies [27]. However, the present study observed an increase in the concentration during later years. The likely source of contamination was ^137^C in the soil and tree litter absorbed by the roots [28], resulting in bioaccumulation of radiocesium after many years. This finding should be highlighted for its increasing radioactivity concentration among a non-high-risk radiation food category. Hence, as a precautionary measure, monitoring the local foods must not be overlooked and it is an essential effort towards keeping the awareness of radiation at raised levels.

Edible wild plants and mushrooms were both consistently contaminated, with samples frequently showing radioactivity > 100 Bq/kg. As shown in Figure 1, these two food categories contain many samples that tested over 1000 Bq/kg. Similar results were obtained in Kawauchi village located 20–30 km west of the FDNPP, where 81.2% of wild mushroom samples had radiocesium levels > 100 Bq/kg [29].

The “others” category included a variety of food samples. Food samples such as meat, honey, and eggs were inspected. This category resulted in a constant median around 100 Bq/kg and an annual average of over 100 Bq/kg. This is possibly from inspecting wild boar meat, one of the most highly contaminated food products, as well as one of the most frequently sampled foods in the “others”. Despite the high contamination risk, hunting and consuming wild boar meat remains a common practice by residents of Nihonmatsu City. The high radiocesium levels in wild boars may be attributed to their habit of ingesting soil attached to their food. It is likely that wild boars were consuming a variety of foods, including plants, mushrooms, and small animals from contaminated areas. A study measuring radioactivity in wild boar meat at different locations around Chernobyl from 2002 to 2011 also found that one of the surveyed areas exceeded the regulation limit multiple times [30]. Considering eating wild boar meat is a common practice, the residents of Nihonmatsu who often hunt may be at risk of internal contamination.

In contrast, radioactivity was not detected or was <20 Bq/kg in at least 97% of inspected potato and vegetable samples. These food groups and other cultivated plants harvested from summer to autumn are planted as seeds and seedlings each year. Typically, radiocesium persisting on the soil surface and circulating in the forest can contaminate these crops and the seeds. However, tilling the soil for cultivation disturbs radiocesium in soil, thereby reducing the amount that migrates into the plants [31]. The findings are also consistent with a previous study showing that the proportion of contaminated rice (>100 Bq/kg), the main staple food of the Japanese diet, decreased from 2011 to 2013 [32].

This study had several limitations. From its observational nature, it is difficult to make any inferences regarding the observed trends in contamination levels. Additionally, Nihonmatsu City residents may have perceived over or under awareness of risk from radioactivity in their food products. Residents who voluntarily offered food samples for inspection may be more cautious of the foods they consume, whilst other residents may not have actively monitored their food from indifference or a lack of awareness of food contamination. Owing to time and fading concern, risk perception may be fading among many residents on contaminated food products. Similarly, after the Chernobyl accident, many people continued to consume locally produced food, possibly because of indifference to warnings regarding the potential harm from internal radiation [33]. Contrarily, mothers and older adults, or retired people, continued to exhibit higher levels of concern about food safety [34]. Furthermore, this study collected data from government-facilitated monitoring centers. This led to excluding data from other resources, such as citizen-run and commercially-run centers, which may examine products for different purposes. Finally, the discrepancies in the measured radiocesium levels from the monitoring instruments cannot be discounted.

## 5. Conclusions

The present study evaluated local food products in Nihonmatsu City of Fukushima Prefecture over six years to raise awareness of internal contamination risk. Although the contamination levels of most foods were within the provisional regulation limits, monitoring the radioactivity within local food products has importance. Given the declining number of inspected products, it is a concern that radiocesium levels may exceed the provisional regulation limit among the local foods for home consumption. While certain food categories such as mushrooms and edible wild plants are still considered high-risk, promptly continuing local food monitoring is the only way to provide food safety even across non high-risk categories.

The findings of this study provide evidence of the importance of continued monitoring of locally grown foods in Fukushima Prefecture, which were impacted by the FDNPP accident. There is an increase in risk of radiation exposure due to the possible decline of citizens’ awareness, accompanied by the decrease of interest in contaminated food. It also highlights that food producers and distributors have similar problems. Therefore, the recommendations include having residents avoid consuming wild animals and plants, and for the continuation of food monitoring to eliminate the risk of internal radiation exposure, with attention to high-risk foods for overall community health. To obtain consumers trust in food safety, efforts should be made at the national, prefectural, and municipal governmental levels to disseminate information about food monitoring.

## Figures and Tables

**Table 1:** Summary of food samples collected in Nihonmatsu City (2011-2017).

Food Group	Potatoes *n* (%)	Vegetables *n* (%)	Grains *n* (%)	Beans *n* (%)	Fruits *n* (%)	Seeds *n* (%)	Edible Wild Plants *n* (%)	Mushrooms *n* (%)	Processed Foods *n* (%)	Other *n* (%)	Total
**2011**	277 (16.6)	534 (31.9)	487 (29.1)	168 (10)	124 (7.4)	5 (0.3)	2 (0.1)	8 (0.5)	60 (3.6)	7 (0.4)	1672
**2012**	2122 (11.1)	8183 (42.9)	1342 (7.0)	1532 (8.0)	2250 (11.8)	518 (2.7)	1626 (8.5)	678 (3.6)	673 (3.5)	139 (0.7)	19,063
**2013**	1041 (9.3)	4974 (44.3)	245 (2.2)	602 (5.4)	1732 (15.4)	276 (2.5)	1495 (13.3)	301 (2.7)	471 (4.2)	101 (0.9)	11,238
**2014**	662 (8.8)	3019 (40.1)	77 (1.0)	380 (5.1)	1117 (14.9)	248 (3.3)	1423 (18.9)	207 (2.8)	255 (3.4)	133 (1.8)	7521
**2015**	647 (8.9)	3220 (44.2)	34 (0.5)	370 (5.1)	1196 (16.4)	324 (4.4)	925 (12.7)	163 (2.2)	262 (3.6)	147 (2.0)	7288
**2016**	544 (9.1)	3069 (51.2)	18 (0.3)	156 (2.6)	662 (11.0)	199 (3.3)	946 (15.8)	94 (1.6)	187 (3.1)	122 (2.0)	5997
**2017**	363 (8.6)	2137 (50.9)	13 (0.3)	146 (3.5)	507 (12)	166 (3.9)	559 (13.3)	59 (1.4)	147 (3.5)	97 (2.3)	4194

**Table 2:** Comparison of radiocesium detection rates by food group.

Potatoes	<ND (%)	≤50 Bq/kg (%)	≤100 Bq/kg (%)	100 Bq/kg < (%)	Vegetables	<ND (%)	≤50 Bq/kg (%)	≤100 Bq/kg (%)	100 Bq/kg < (%)
**2011**	91.3	7.9	0.7	0.0	**2011**	92.9	4.9	1.5	0.7
**2012**	96.6	3.1	0.3	0.0	**2012**	94.4	4.1	1.0	0.5
**2013**	98.1	1.8	0.0	0.1	**2013**	97.0	2.4	0.4	0.2
**2014**	99.2	0.8	0.0	0.0	**2014**	98.2	1.5	0.3	0.0
**2015**	99.1	0.9	0.0	0.0	**2015**	98.9	1.0	0.1	0.0
**2016**	99.3	0.6	0.2	0.0	**2016**	99.0	0.8	0.2	0.0
**2017**	99.2	0.6	0.3	0.0	**2017**	99.0	0.9	0.1	0.0
**Total**	98	2	0	0	**Total**	97	2	1	0
**Grains**	**<ND (%)**	**≤50 Bq/kg (%)**	**≤100 Bq/kg (%)**	**100 Bq/kg < (%)**	**Beans**	**<ND (%)**	**≤50 Bq/k (%)**	**≤100 Bq/kg (%)**	**100 Bq/kg < (%)**
**2011**	79.1	12.7	5.7	2.5	**2011**	20.2	50.6	19.6	9.5
**2012**	84.1	11.7	3.4	0.8	**2012**	55.4	29.1	9.7	5.7
**2013**	89.8	6.9	2.9	0.4	**2013**	74.6	18.4	6.0	1.0
**2014**	92.2	6.5	0.0	1.3	**2014**	89.5	9.2	0.8	0.5
**2015**	97.1	2.9	0.0	0.0	**2015**	93.5	5.1	1.4	0.0
**2016**	88.9	5.6	5.6	0.0	**2016**	95.5	3.2	0.6	0.6
**2017**	100.0	0.0	0.0	0.0	**2017**	97.3	1.4	1.4	0.0
**Total**	84	11	4	1	**Total**	69	21	7	3
**Fruits**	**<ND (%)**	**≤50 Bq/kg (%)**	**≤100 Bq/kg (%)**	**100 Bq/kg < (%)**	**Seeds**	**<ND (%)**	**≤50 Bq/kg (%)**	**≤100 Bq/kg (%)**	**100 Bq/kg < (%)**
**2011**	8.1	17.7	33.9	40.3	**2011**	40.0	40.0	20.0	0.0
**2012**	47.8	33.5	12.5	6.2	**2012**	16.8	27.0	33.4	22.8
**2013**	80.7	15.2	3.1	1.0	**2013**	38.8	38.4	13.4	9.4
**2014**	93.2	5.9	0.9	0.0	**2014**	61.3	27.4	7.7	3.6
**2015**	97.0	2.8	0.2	0.0	**2015**	66.0	19.1	6.8	8.0
**2016**	99.1	0.6	0.3	0.0	**2016**	73.4	9.5	8.0	9.0
**2017**	100.0	0.0	0.0	0.0	**2017**	74.1	12.0	6.0	7.8
**Total**	77	15	5	3	**Total**	48	24	16	12
**Edible wild plants**	**<ND (%)**	**≤50 Bq/kg (%)**	**≤100 Bq/kg (%)**	**100 Bq/kg < (%)**	**Mushrooms**	**<ND (%)**	**≤50 Bq/kg (%)**	**≤100 Bq/kg (%)**	**100 Bq/kg < (%)**
**2011**	50.0	0.0	0.0	50.0	**2011**	0.0	0.0	12.5	87.5
**2012**	31.5	18.9	22.8	26.7	**2012**	8.0	4.1	7.2	80.7
**2013**	44.3	25.6	13.7	16.4	**2013**	10.0	5.0	10.0	75.1
**2014**	56.7	21.5	11.4	10.4	**2014**	11.6	5.3	7.7	75.4
**2015**	61.9	18.5	8.1	11.5	**2015**	14.1	4.3	9.8	71.8
**2016**	63.6	19.0	8.2	9.1	**2016**	21.3	11.7	7.4	59.6
**2017**	69.6	15.0	7.2	8.2	**2017**	28.8	16.9	13.6	40.7
**Total**	51	21	13	15	**Total**	11	5	8	75
**Processed foods**	**<ND (%)**	**≤50 Bq/kg (%)**	**≤100 Bq/kg (%)**	**100 Bq/kg < (%)**	**Others**	**<ND (%)**	**≤50 Bq/kg (%)**	**≤100 Bq/kg (%)**	**100 Bq/kg < (%)**
**2011**	18.3	16.7	28.3	36.7	**2011**	28.6	0.0	42.9	28.6
**2012**	38.6	19.5	17.4	24.5	**2012**	24.5	18.0	19.4	38.1
**2013**	62.0	26.1	8.9	3.0	**2013**	28.7	18.8	17.8	34.7
**2014**	71.4	16.1	6.3	6.3	**2014**	30.8	16.5	21.8	30.8
**2015**	75.6	14.9	4.6	5.0	**2015**	30.6	18.4	7.5	43.5
**2016**	79.7	11.2	4.3	4.8	**2016**	34.4	9.0	6.6	50.0
**2017**	89.8	6.1	2.0	2.0	**2017**	35.1	19.6	14.4	30.9
**Total**	60	18	10	12	**Total**	30	16	15	38

## Supplementary Materials

The following are available online at http://www.mdpi.com/1660-4601/15/10/2289/s1. Figure S1: Radiocesium detection rates by detection level for each food group.
