# Supplementary figures and images for: The Knowledge and Awareness for Radiocesium Food Monitoring after the Fukushima Daiichi Nuclear Accident in Nihonmatsu City, Fukushima Prefecture
Source: Int J Environ Res Public Health. 2018 Oct 18;15(10):2289. doi: 10.3390/ijerph15102289 (PMC6210092; doi:10.3390/ijerph15102289)

Supplementary Figure 1. Radiocesium detection rates of each year by food group

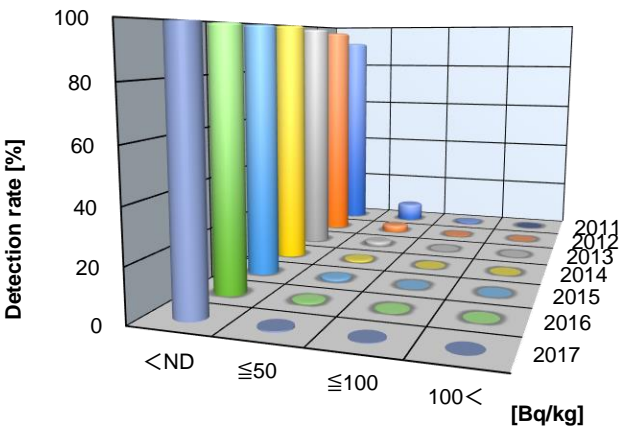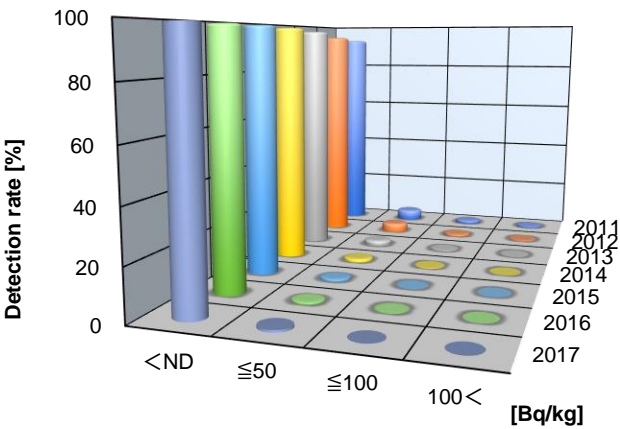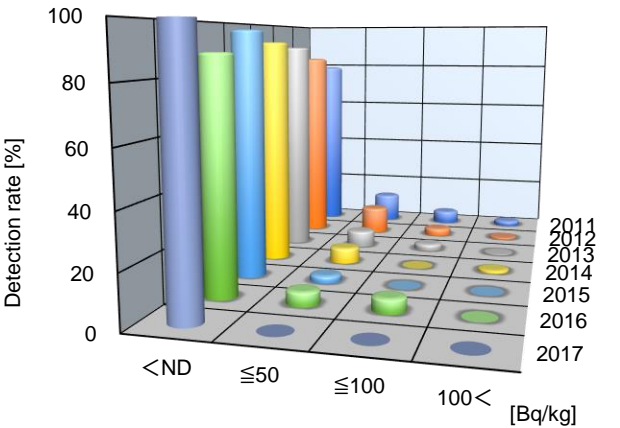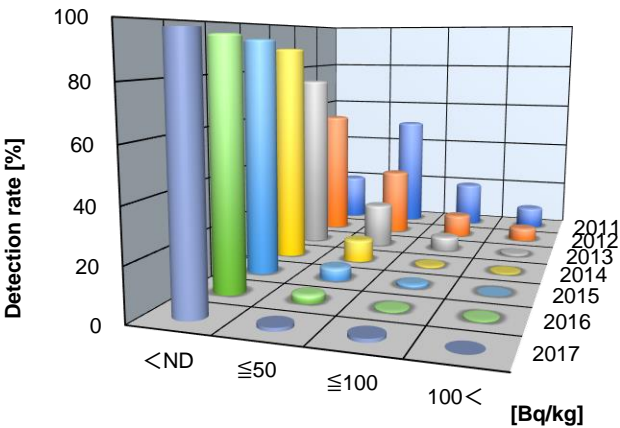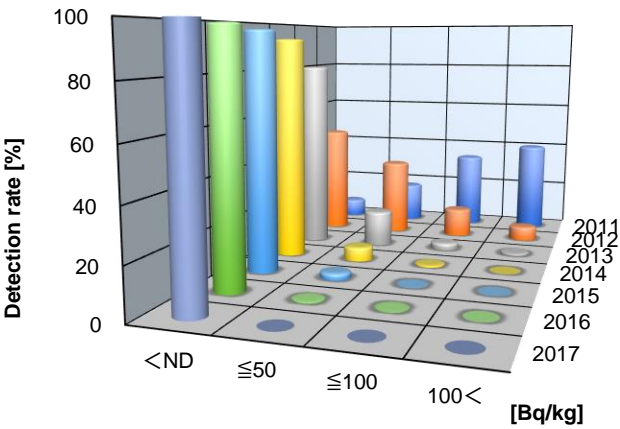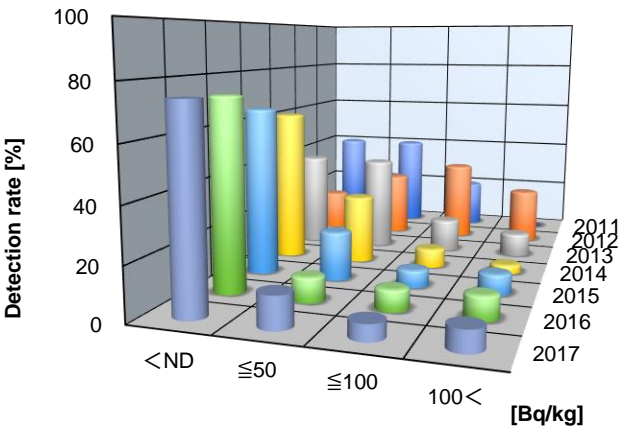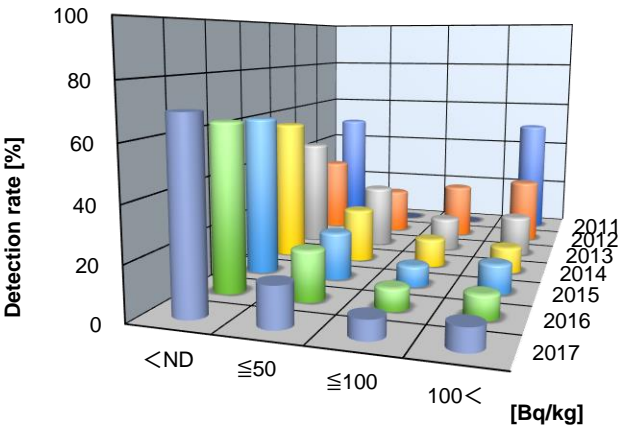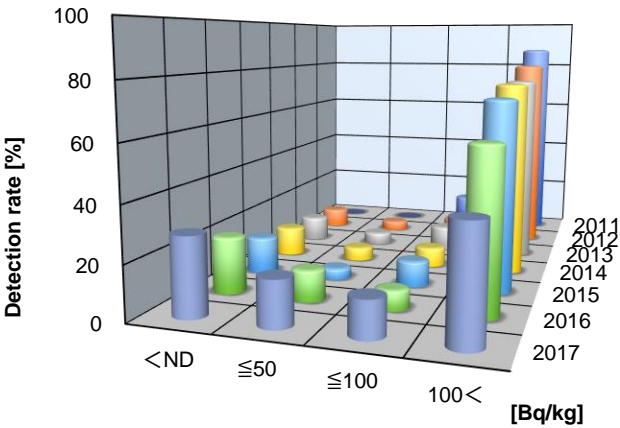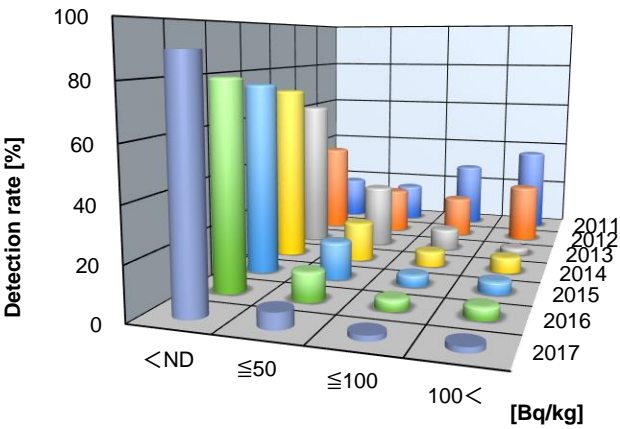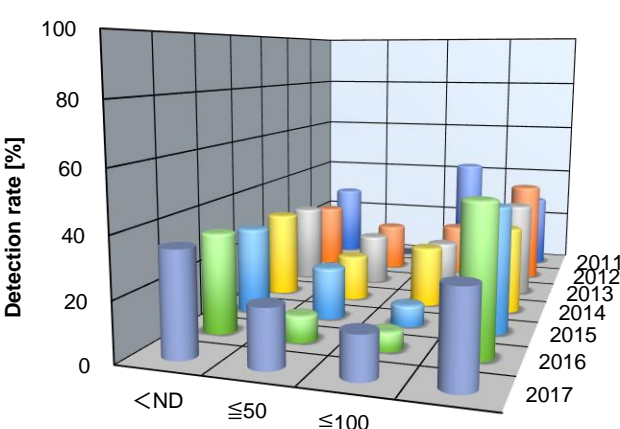

Supplement: Supplementary file 1 [file ijerph-15-02289-s001.pdf]
